# Rim enhancement of pancreatic ductal adenocarcinoma: investigating the relationship with DCE-MRI-based radiomics and next-generation sequencing

**DOI:** 10.3389/fonc.2024.1304187

**Published:** 2024-03-08

**Authors:** Moon Hyung Choi, Seung Bae Yoon, Young Joon Lee, Eun Sun Jung, Seongyong Pak, Dongyeob Han, Dominik Nickel

**Affiliations:** ^1^ Department of Radiology, Eunpyeong St. Mary’s Hospital, College of Medicine, The Catholic University of Korea, Seoul, Republic of Korea; ^2^ Department of Internal Medicine, Eunpyeong St. Mary’s Hospital, College of Medicine, The Catholic University of Korea, Seoul, Republic of Korea; ^3^ Department of Hospital Pathology, Eunpyeong St. Mary’s Hospital, College of Medicine, The Catholic University of Korea, Seoul, Republic of Korea; ^4^ Research Collaboration, Siemens Healthineers Ltd., Seoul, Republic of Korea; ^5^ MR Applications Predevelopment, Siemens Healthcare GmbH, Erlangen, Germany

**Keywords:** pancreatic cancer, radiomics, genetics, dynamic contrast-enhanced imaging, magnetic resonance imaging

## Abstract

**Purpose:**

To identify the clinical and genetic variables associated with rim enhancement of pancreatic ductal adenocarcinoma (PDAC) and to develop a dynamic contrast-enhanced (DCE) MRI-based radiomics model for predicting the genetic status from next-generation sequencing (NGS)

**Materials and methods:**

Patients with PDAC, who underwent pretreatment pancreatic DCE-MRI between November 2019 and July 2021, were eligible in this prospective study. Two radiologists evaluated presence of rim enhancement in PDAC, a known radiological prognostic indicator, on DCE MRI. NGS was conducted for the tissue from the lesion. The Mann-Whitney U and Chi-square tests were employed to identify clinical and genetic variables associated with rim enhancement in PDAC. For continuous variables predicting rim enhancement, the cutoff value was set based on the Youden’s index from the receiver operating characteristic (ROC) curve. Radiomics features were extracted from a volume-of-interest of PDAC on four DCE maps (K^trans^, K_ep_, V_e_, and iAUC). A random forest (RF) model was constructed using 10 selected radiomics features from a pool of 392 original features. This model aimed to predict the status of significant NGS variables associated with rim enhancement. The performance of the model was validated using test set.

**Results:**

A total of 55 patients (32 men; median age 71 years) were randomly assigned to the training (n = 41) and test (n = 14) sets. In the training set, KRAS, TP53, CDKN2A, and SMAD4 mutation rates were 92.3%, 61.8%, 14.5%, and 9.1%, respectively. Tumor size and KRAS variant allele frequency (VAF) differed between rim-enhancing (n = 12) and nonrim-enhancing (n = 29) PDACs with a cutoff of 17.22%. The RF model’s average AUC from 10-fold cross-validation for predicting KRAS VAF status was 0.698. In the test set comprising 6 tumors with low KRAS VAF and 8 with high KRAS VAF, the RF model’s AUC reached 1.000, achieving a sensitivity of 75.0%, specificity of 100% and accuracy of 87.5%.

**Conclusion:**

Rim enhancement of PDAC is associated with KRAS VAF derived from NGS-based genetic information. For predicting the KRAS VAF status in PDAC, a radiomics model based on DCE maps showed promising results.

## Introduction

Pancreatic cancer is the fourth most common cancer with the lowest 5-year relative survival rate (11%) in the United States (1). MRI offers higher soft-tissue contrast, which is helpful for detecting and characterizing small lesions in the pancreas and liver (2, 3). A prior study attempted to find radiological findings that would predict clinical outcome, and rim enhancement of pancreatic ductal adenocarcinoma (PDAC) on MRI was an independent predictor of poor outcome in patients who received surgery (4). Lesions with rim enhancement showed more aggressive histologic tumor grades, fewer visible acini, and more necrosis inside the tumor than lesions without rim enhancement.

Although multiphase MRI is commonly utilized for pancreatic imaging, dynamic contrast-enhanced (DCE) MRI with short temporal resolution (< 10 seconds) has been investigated. Previous research found that DCE MRI findings differed significantly between pancreatic tumors and normal pancreas or benign disease (5–8). The tumor’s characteristics are expected to be quantitatively analyzed using DCE MRI parameters which are correlated to pathological findings such as microvascular density or fibrosis (8–11). Additionally, DCE MRI parameters are different depending on the therapeutic response in PDAC patients (12, 13).

Radiomics is used to extract high-dimensional features and to quantitatively assess details on radiological images that cannot be seen visually (14, 15). In radiomics, features are selected based on predefined mathematical calculations that explain the relationships between signal intensities in pixels. A machine learning algorithm is used to choose several important features from hundreds of available ones and to construct a prediction model. Multiple studies have been performed to discover key radiomics features or to build radiomics models to predict pathologic characteristics or patient outcomes in oncology (16, 17). Radiomics in the pancreas has been used to differentiate pancreatic lesions from the normal pancreas, classify pancreatic masses, and predict therapeutic response or prognosis (18–20). Radiogenomics is a specialized application that connects radiomics to genetic data (21, 22). DCE MRI, however, has not been employed for radiomics or radiogenomics research in the pancreas. We anticipated that the quantitative analysis using radiomics in DCE MRI, which might reflect the histologic features of the tumor, could potentially have a correlation with qualitative MRI findings such as rim enhancement or genetic characteristics. If the quantitative analysis of MRI is related to genetic prognostic factors, it is expected that MRI variables could serve as potential prognostic factors. Therefore, the purpose of this study was to identify the clinical and genetic variables associated with rim enhancement of PDAC as well as to develop and test a radiomics model based on DCE MRI parametric maps for predicting the status of important genetic factors.

## Materials and methods

### Patients

Our hospital’s institutional review board approved this prospective study, and informed consent was obtained from all participants.

Patients diagnosed with PDAC at our institution after July 2019, and had their diagnosis pathologically confirmed via biopsy or surgery, were eligible. From this group, we only included those who underwent a pre-treatment pancreas MRI that would be used for analysis. We set our target study participant count to 60 based on precedent. This decision was informed by previous DCE MRI studies on PDAC, where participant numbers ranged from 14 to 58, especially considering the unpredictability of correlating DCE MRI results with genetic information (8, 10, 11, 23–25). Exclusion criteria were as follows: (a) no pancreatic MRI prior to treatment; (b) pancreatic MRI that did not include DCE MRI; (c) pancreatic MRI at other institutions; and (d) refusal to participate in the study. Clinical data from electronic medical records were collected, including age, sex, initial carbohydrate antigen (CA) 19-9 level, and clinical staging. The patients were randomly assigned into two groups, i.e., training and test sets, in a 3:1 ratio.

### MRI acquisition

A 3T MRI scanner (MAGNETOM Vida, Siemens Healthcare, Erlangen, Germany) with a 30-channel surface coil and a 32-channel or 72-channel spine coil was utilized for all MRI examinations. A power injector operating used to deliver 0.1 mmol/kg gadoterate meglumine (Dotarem, Guerbet, Paris, France) followed by a 20-mL saline flush for DCE MRI. The temporal resolution of DCE MRI was 13.5 seconds for the first two images, 8.4 seconds for 180 seconds, and 13.5 seconds for the remaining 121 seconds. The MRI sequences and parameters are summarized in **Supplementary Table E1**. Pharmacokinetic maps were generated from DCE MRI after automatic motion correction and registration using a commercially available program (MR Tissue4D in Syngo.via VB40B, Siemens Healthcare): volume transfer constant (K^trans^), reverse reflux constant (K_ep_), extravascular extracellular volume fraction (V_e_). The initial area under the curve (iAUC) was measured for the first 60 seconds. The arterial input function was chosen by having the smallest chi2 value as supplied by the program.

### Image analysis

MR examinations were reviewed independently by two abdominal radiologists. They measured tumor size based on DCE MRI referring to all other sequences. They also evaluated whether the tumor had rim enhancement on DCE MRI images, as defined in a previous study: irregular ring-like enhancement with a relatively hypo-enhancing central area (4). The discordant results were solved by consensus, and the final decision was regarded as the gold standard of tumor size and rim enhancement.

### Next generation sequencing (NGS)

An expert pathologist reviewed the hematoxylin-eosin-stained slides to determine the cancer area and normal pancreas tissue as well as the existence of an adequate amount of tissue for NGS. The Oncomine Comprehensive Assay Plus panel (Thermo Fisher Scientific, USA) was used for NGS, which targeted 411 genes of solid tumors. Tier I or II genetic alterations were detected using standards and guidelines for the assessment and reporting of sequence variants in cancer (26). The thresholds of variant allele frequency for hotspot variants, single nucleotide variants (SNVs), and insertions and deletions (indels) were ≥ 4%, ≥ 5% and ≥ 5%, respectively. Copy number variation ≥ 4 was considered a gain (amplification), and a variation < 0.7 was considered a loss (deletion).

### Tumor segmentation

One radiologist with 10 years of experience in abdominal imaging performed 3D tumor segmentation on the pancreatic phase of DCE MRI by referring to all available MR images. Volume of interest (VOI) segmentation was performed manually on all axial images of the tumor, using open source software ITK-SNAP, version 3.8.0 (http://www.itksnap.org/) (27). If a patient had multiple cancer lesions, tumor segmentation was performed on the largest tumor. To assess intraobserver agreement, the radiologist performed tumor segmentation again for each patient more than a month after completing the first segmentation.

### Radiomics feature extraction

Software for radiomics analysis (Syngo. Via Frontier, Version 1.2.2; Siemens Healthineers) was used (28). This software package was developed based on the PyRadiomics library, version 3.0.1 (https://github.com/Radiomics/pyradiomics) and scikit-learn machine learning library (https://scikit-learn.org/stable/modules/generated/sklearn.ensemble.RandomForestClassifier.html). Four DCE parametric maps were simultaneously loaded into the software with a segmentation mask. MR images were resampled using B-Spline interpolation at a spatial resolution of 1 × 1 × 1 mm3. The bin width was set as 25 to make a histogram of discretization of the image gray levels. On each DCE parametric map, 110 original features were extracted from a VOI. They included 18 first-order features, 17 shape features and 75 texture features (gray level dependence matrix [GLDM], gray level co-occurrence matrix [GLCM], gray level run length matrix [GLRLM], gray level size zone matrix [GLSZM], and neighboring gray tone difference matrix (NGTDM) features). The software generated a cluster map to show associations between the identified clusters of patients and features using the Ward variance minimization algorithm to calculate the cluster distances (**Supplementary Figure E1**).

### Feature selection, radiomics model development and testing

Radiomics features from four DCE parametric maps were integrated. In the training set, features having an intraclass correlation coefficient (ICC) of less than 0.75 between two VOIs were removed (29). Radiomics features were reduced to a maximum of 10 features using the classic minimum redundancy maximum relevance (mRMR) method based on the R^2^ difference. The algorithm selects the most relevant features for target classification while minimizing feature redundancy. Using the selected features, a random forest (RF) model for predicting the significant genetic factor was built. The model was optimized using tenfold cross-validation, and the average area under the receiver operating characteristic curve (AUC), sensitivity, specificity and accuracy were calculated. The model was optimized using tenfold cross-validation and validated with a test set.

### Statistical analysis

The Kolmogorov-Smirnov test was performed to evaluate the normality of the continuous clinical variables, including age, tumor size and CA19-9 level, and variant allele frequency (VAF) of four most common mutations identified by NGS. Cohen’s kappa value and ICC were used to assess interobserver agreement for rim enhancement and tumor size measurement. The Dice similarity coefficient was employed to assess spatial agreement between two sets of VOIs of PDAC. Mann-Whitney U and Chi-square tests were used to compare clinical and genetic factors between training and test sets as well as between tumors with and without rim enhancement. The correlations among the significant factors were evaluated using the Spearman correlation coefficient. Receiver operating characteristic (ROC) curve analysis was assessed the discriminative ability of continuous variables from NGS in predicting the presence of rim enhancement. Youden’s index, applied to the training set, determined the cutoff values for significant factors linked to rim enhancement.

A radiomics model was built utilizing radiomics features from DCE parametric maps to predict the status of the significant genetic factor. The sensitivity, specificity, accuracy, and AUC of the radiomics model were calculated in the test set and training set. Statistical analyses were performed using SPSS software version 23.0 (IBM, Armonk, NY, USA) and GraphPad Prism version 8.0 (GraphPad Software, Inc., La Jolla, CA, USA). *P* <.05 was considered statistically significant.

## Results

### Patients

**Figure 1:**
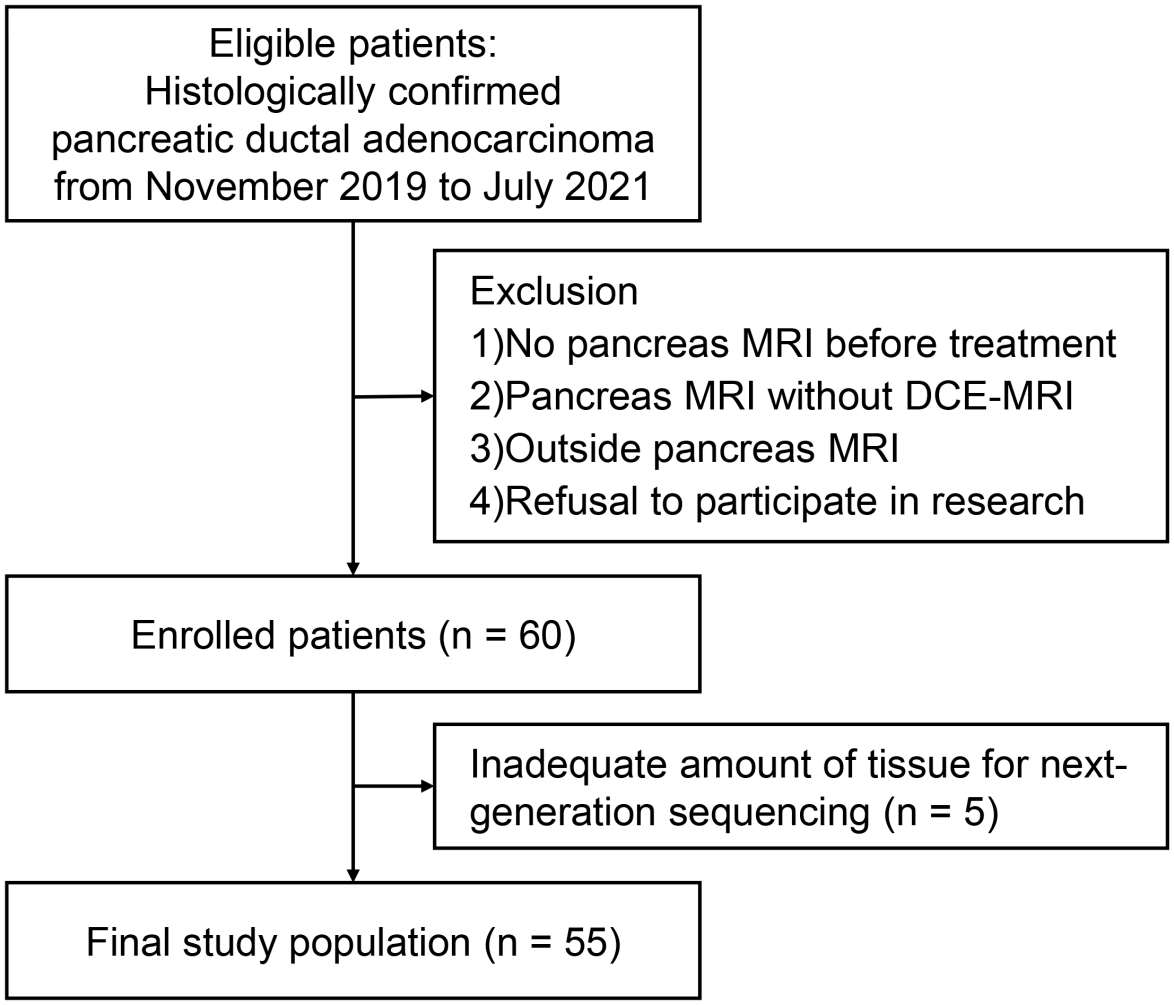
Flow diagram of study participants.

From November 2019 to July 2021, 60 patients consented to participate in this study. Five patients were excluded due to unavailable NGS results because of insufficient amounts of tissue (**Figure 1**). A total of 55 patients (32 men, median age 71 years, interquartile range [IQR], 66–77]) were included. The median CA19-9 level was 470.2 U/mL (IQR, 49.3–2972.0 U/mL). Although two patients had two pancreatic cancer lesions, only the largest lesion was included in the analysis. Resectable, borderline resectable, locally advanced, and metastatic PDAC were diagnosed in 16 (29.1%), 3 (5.5%), 7 (12.7%) and 29 (52.7%) patients, respectively. Histological tumor grading was available for 29 patients: 8 had well-differentiated tumors, 18 had moderately differentiated tumors, and 3 had poorly differentiated tumors. Surgery was performed in 14 patients. In the training and test sets, 41 and 14 patients were randomly assigned. **Table 1** summarizes the baseline characteristics of the patients. There were no statistically significant differences in any clinical factor between the training and test sets.

**Table 1:** Clinical characteristics in the training and test sets.

Characteristics	Training set (n = 41)	Test set (n = 14)	*P* value
Age (years)	72.0 (66.0–77.5)	69.0 (66.0–77.3)	0.756
Male patient	23 (56.1%)	9 (64.3%)	0.416
Body weight (kg)	60.0 (50.0–68.5)	61.5 (52.3–68.5)	0.977
CA 19-9 (U/mL)	421.1 (60.5–3184.5)	664.2 (20.9–2797.5)	0.885
Tumor size (cm)	3.6 (2.4–5.0)	2.8 (2.3–4.2)	0.422
Tumor location			
Head	20 (48.8%)	6 (42.9%)	0.704
Body to tail	21 (51.2%0)	8 (57.1%)	
Clinical staging			
Resectable PDAC	10 (24.4%)	6 (42.9%)	0.291
Borderline resectable PDAC	2 (4.9%)	1 (7.1%)	
Locally advanced PDAC	7 (17.1%)	0 (0%)	
Metastatic PDAC	22 (53.7%)	7 (50.0%)	

Data are presented as median (interquartile range) or number (%).

CA, Carbohydrate antigen.

### Image analysis and segmentation

The median tumor size in all patients was 3.5 cm (IQR, 2.3–5.0 cm). The ICC for the size measurement between two readers was 0.900. The two radiologists classified 19 and 17 tumors as positive rim enhancement, respectively (κ = 0.670). Following the resolution of the disagreement, 18 patients (32.7%) were categorized as having tumors with rim enhancement, including 12 patients in the training set and 6 patients in the test set. In all cases, the Dice similarity coefficient between the two sets of VOIs in all patients was 0.760.

### Clinical and genetic factors between tumors according to rim enhancement

In the training set, tumors with rim enhancement were significantly larger than tumors without rim enhancement (*P* = 0.021) (**Table 2**). Other clinical factors were not different according to rim enhancement. As a result of NGS, a wide variety of genetic mutations were discovered (**Supplementary Figure E2**). We evaluated the four most common mutations in PDAC. KRAS, TP53, CDKN2A, and SMAD4 mutation rates were 92.3%, 61.8%, 14.5%, and 9.1%, respectively. The presence or absence of these mutations was not different according to tumor rim enhancement. The VAF of KRAS mutation was significantly higher in tumors with rim enhancement than in others. The VAFs of other mutations did not differ between the two groups. Spearman correlation test showed that tumor size and KRAS VAF were not correlated (ρ = 0.275, *P* = 0.082).

**Table 2:** Differences in clinical characteristics and genetic information according to rim enhancement in the training set.

Parameters	Rim enhancement (n = 12)	No rim enhancement (n = 29)	*P* value
Clinical characteristics			
Age (years)	71.0 (64.3–79.3)	72.0 (66.0–77.5)	0.767
Male patient	7 (58.3%)	16 (55.2%)	0.853
Body weight (kg)	71.0 (64.3–67.3)	60.0 (50.0–70.0)	0.372
CA 19-9 (U/mL)	1993.6 (99.9–16911.5)	261.8 (43.3–1265.9)	0.176
Tumor size (cm)	4.8 (4.2–6.0)	3.3 (2.2–4.9)	0.021
Clinical stage			
Resectable PDAC	1 (8.3%)	9 (31.0%)	0.221
Borderline resectable PDAC	1 (8.3%)	1 (3.4%)	
Locally advanced PDAC	1 (8.3%)	6 (20.7%)	
Metastatic PDAC	9 (75.0%)	13 (44.8%)	
Mutation profile			
KRAS mutant	12 (100%)	25 (86.2%)	0.235
KRAS VAF (%)	27.3 (20.8–35.7)	13.2 (6.3–25.4)	0.008
TP53 mutant	9 (75.0%)	16 (55.2%)	0.236
TP53 VAF (%)	13.0 (0–40.1)	10.5 (0–21.8)	0.488
CDKN2A mutant	2 (16.7%)	4 (13.8%)	0.813
CDKN2A VAF (%)	0 (0–0)	0 (0–0)	0.956
SMAD4 mutant	1 (8.3%)	3 (10.3%)	0.843
SMAD4 VAF (%)	0 (0–0)	0 (0–0)	0.944

Data are presented as median (interquartile range) or number (%).

PDAC, pancreatic ductal adenocarcinoma; CA, Carbohydrate antigen; VAF, variant allele frequency.

### The cutoff value for positive rim enhancement

Two factors (tumor size and KRAS VAF) that exhibited significant differences between tumors with and without rim enhancement were further evaluated with ROC curves. In the training set, the cutoff values of tumor size and KRAS VAF for predicting positive rim enhancement were > 3.9 cm and > 17.22%; they had AUCs of 0.728 and 0.762, respectively. Based on the established cutoff of KRAS VAF, 10 out of the 12 rim-enhancing PDAC cases and 8 out of the 29 nonrim-enhancing PDAC cases were classified with high KRAS VAF. In the test set, the AUCs for the tumor size and KRAS VAF were 0.510 and 0.750, respectively (**Supplementary Figure E3**). According to the KRAS VAF cutoff, patients in the test set were divided into two groups, namely, low KRAS VAF [n = 6] and high KRAS VAF [n = 8].

### Development and testing of the radiomics model

**Figure 2:**
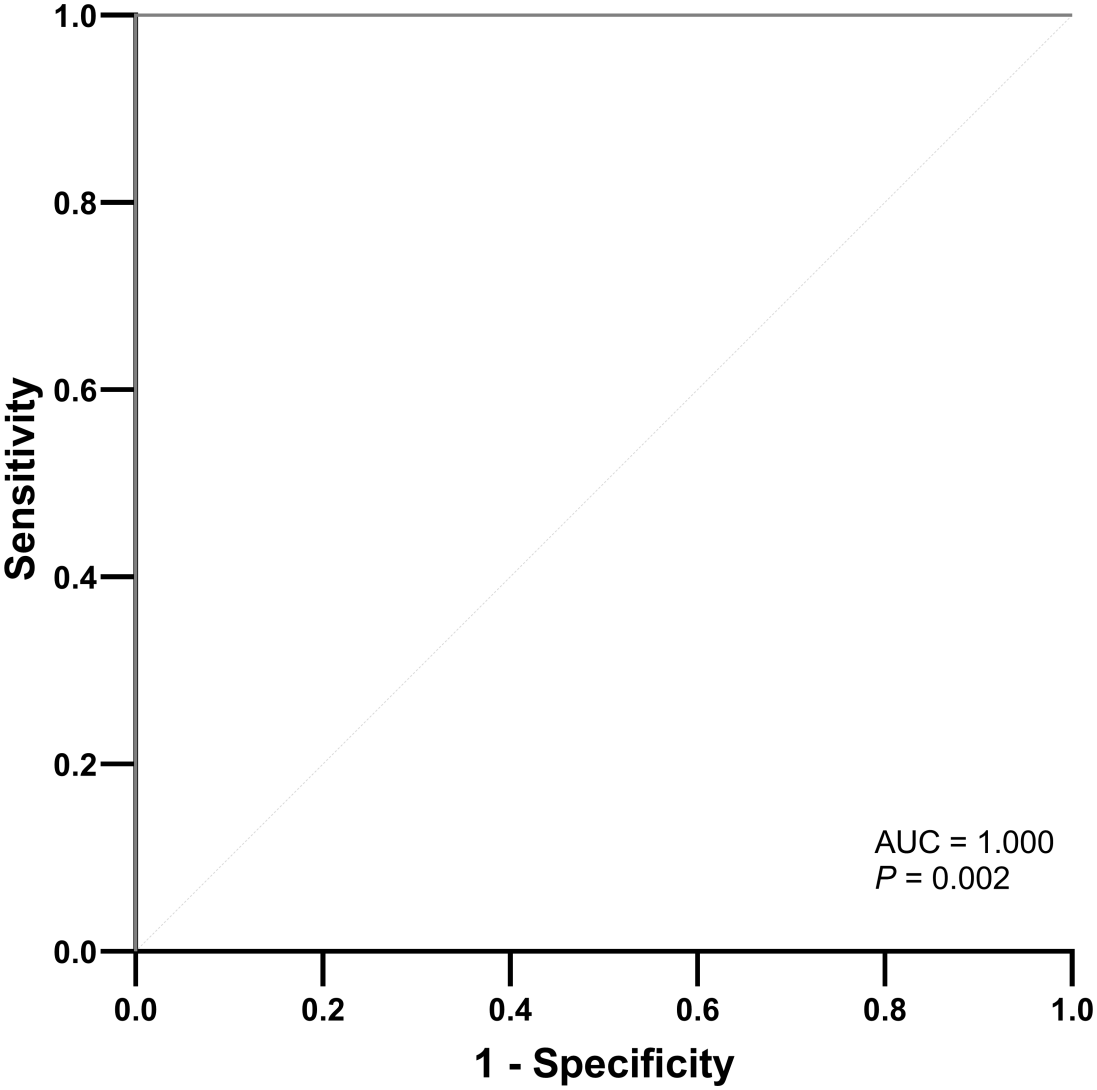
ROC curve of the radiomics model for predicting high KRAS variant allele frequency in the test set.

**Figure 3:**
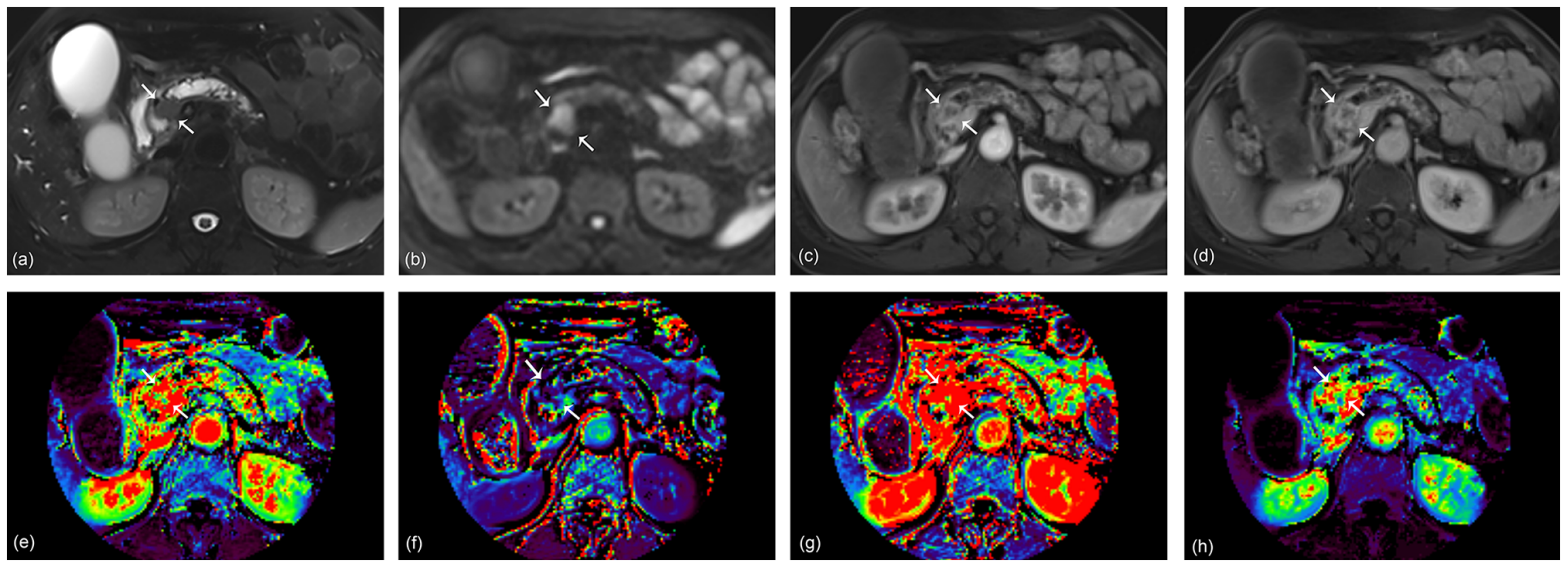
A 68-year-old woman with pancreatic ductal adenocarcinoma in the pancreas head. A 2.3 cm tumor shows high signal intensity on axial T2-weighted image **(A)** and diffusion-weighted image **(B)**. Arterial **(C)** and portal **(D)** phase images of dynamic contrast-enhanced (DCE) MRI show rim enhancement of the tumor. K^trans^ **(E)**, K_ep_ **(F)**, V_e_ **(G)**, and iAUC **(H)** maps are displayed at the level of the tumor. The KRAS variant allele frequency (VAF) of this lesion was 20.6%, and the patient was classified as having a high VAF level. The radiomics model based on DCE parameters predicted the lesion as a high VAF tumor with a probability of 0.59.

**Figure 4:**
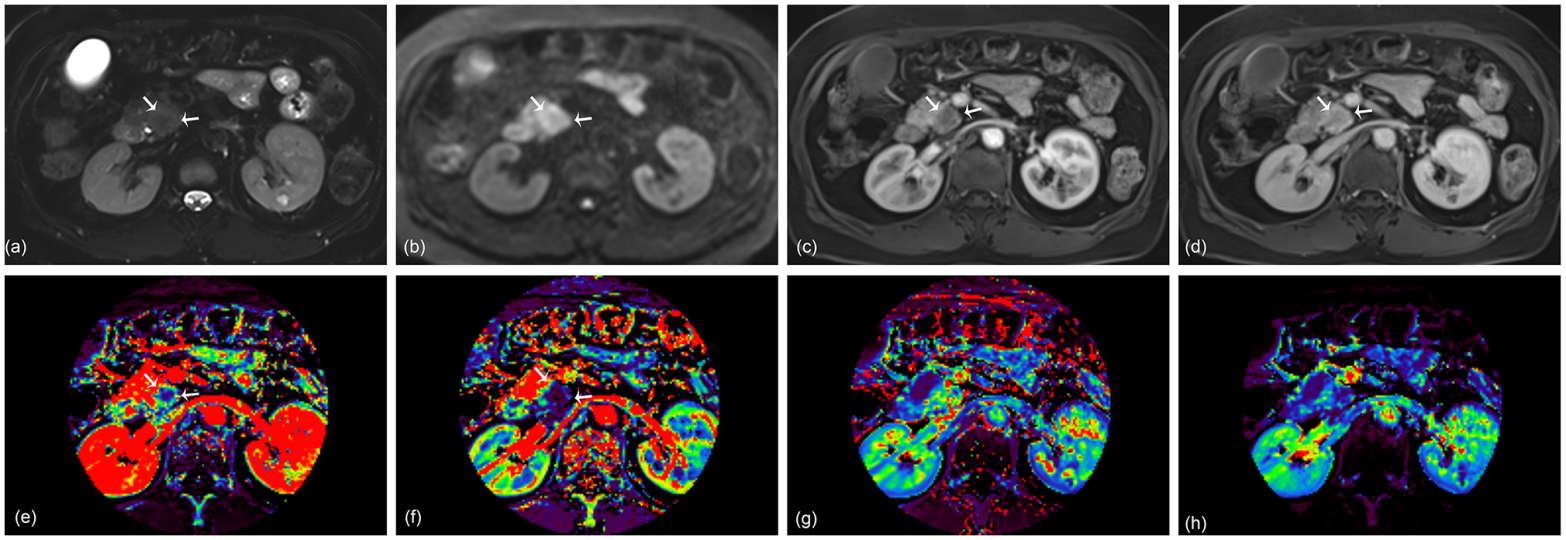
A 67-year-old woman with pancreatic ductal adenocarcinoma in the pancreas head. A 2.4 cm tumor shows high signal intensity on axial T2-weighted image **(A)** and diffusion-weighted image **(B)**. Arterial **(C)** and portal **(D)** phase images of dynamic contrast-enhanced (DCE) MRI show no rim enhancement of the tumor. K^trans^ **(E)**, K_ep_ **(F)**, V_e_ **(G)**, and iAUC **(H)** maps are displayed at the level of the tumor. The KRAS variant allele frequency (VAF) of this lesion was 13.5%, and the patient was classified as having a low VAF level. The probability score of the radiomics model based on DCE parameters was 0.12 and predicted the lesion as a low VAF tumor.

A radiomics model utilizing DCE parameters was developed to predict KRAS VAF status. After excluding 17 features with low ICC from each parametric map, 93 features were selected from each DCE map. Consequently, a total of 372 features were extracted from the four DCE maps. From the training set, the ten most important characteristics for predicting low and high KRAS VAF were chosen (**Supplementary Figure E4**). The average AUC of the radiomics model with 10-fold cross validation was 0.698. The model’s sensitivity, specificity, and accuracy were 66.7%, 82.6% and 75.6%, respectively. In the test set, the AUC of the model was 1.000 (**Figure 2**). The sensitivity, specificity and accuracy of the model were 75.0%, 100% and 87.5%, respectively. The example cases are depicted in **Figures 3**, **4**.

## Discussion

Our study evaluated the clinical and genetic factors that are associated with rim enhancement of PDAC, which has been identified as a predictive imaging feature for postsurgical prognosis (4). Only tumor size and VAF of KRAS mutation were associated with rim enhancement; the presence of any common mutation in PDAC was not associated. We used a machine learning model based on radiomics of DCE MRI to predict low and high KRAS VAFs (cutoff 17.22%). A machine learning model incorporating DCE parametric maps (K^trans^, K_ep_, V_e_, iAUC) produced excellent results with an AUC of 1.000 in the test set that was randomly selected from the entire patient cohort. In this study, we discovered genetic differences in PDAC based on rim enhancement and used DCE MRI radiomics to predict genetic information.

KRAS, TP53, CDKN2A, and SMAD4 are well-known driver mutations in PDAC. Recent advances in NGS technology enable accurate genetic mutation profiling of PDAC even with a small biopsy sample (30). The mutation rates of KRAS/TP53/CDKN2A/SMAD in the entire patient group in our study were consistent with previous results (31, 32). Because PDAC involves a very intricate molecular process, single major genetic alterations have seldom demonstrated therapeutic or prognostic implications in clinical settings. Beyond the presence of mutations, quantitative genetic variant analysis may be necessary to properly assess clinical genomic information in PDAC.

In the current study, KRAS VAF levels were linked to PDAC rim enhancement in MRI. VAF is defined as the percentage of sequence reads in a particular sample that have a certain deoxyribonucleic acid (DNA) variant. According to recent research, patients with higher KRAS VAF exhibited greater tumor cellularity and worse survival outcomes (33, 34). There was no significant relationship between the positivity of KRAS mutation and survival outcome in a recent study on KRAS mutation in resected pancreatic cancer specimens. Rather, inverse relationships of KRAS VAF with survival outcomes were consistently reported across strata of tumor cellularity levels (35). The same study reported that a higher KRAS VAF was associated with a higher frequency of neural/lymphatic invasion, increased tumor cellularity, and decreased inflammatory cellularity. Mechanical evidence from animal models supports our findings, which suggest that a higher rate of KRAS mutation contributes to rapid cancer progression and metastasis (36, 37).

Rim-enhancing PDAC exhibited significantly greater intratumoral necrosis and a higher aggressive grade as well as a significantly worse survival result than nonrim-enhancing PDAC (4). MRI-based intratumoral necrosis, defined as a region with fluid signal intensity and poor contrast enhancement, was correlated with more pathological intratumoral necrosis, higher tumor cellularity and worse clinical outcome in another study (38). As a result, the enhancement pattern may represent the histological features of PDAC as well as the clinical outcome.

In our study, rim-enhancing PDAC exhibited significantly higher levels of KRAS VAF than nonrim-enhancing PDAC. While we did not investigate the direct association between patient survival and high KRAS VAF, our results indicate that the radiologically unfavorable prognostic finding is related to high KRADS VAF, which has been associated with a worse prognosis in prior research (35). However, no relationship was found between the existence of major genetic mutations and the rim enhancement of PDAC. The particular mutation in PDAC may not alter the phenotype on radiological imaging, similar to earlier clinical research in which KRAS mutation was not associated with the patient’s prognosis (35).

Because the enhancement pattern is connected to NGS-based genetic information, quantitative analysis for contrast enhancement utilizing DCE MRI was applied in this study. As rim enhancement indicates varying degrees of enhancement in the peripheral and central areas of the tumor, the mean values of DCE parameters in the entire tumor may not accurately reflect tumor enhancement. Therefore, we used radiomics analysis of DCE parametric maps to predict KRAS VAF status in PDAC. In a prior study, radiomics models based on arterial and portal phase contrast-enhanced MRI were developed to predict Mucin 4 expression levels (39). However, no radiomics study of DCE parametric maps has been performed in PDAC. To build a radiomics model, we combined three DCE parametric maps (K^trans^, K_ep_, and V_e_ maps) and an iAUC map. Furthermore, to simplify the radiomics model, we used only the original features, excluding filtered features from processes like wavelet and Laplacian of Gaussian filtering. The radiomics model with 10 features selected from four different maps showed excellent results in predicting high and low KRAS VAF tumors in the test set with an AUC of 1.000. Although radiomics features may not be directly interpreted as classical image findings, we can infer their implications. Among the 10 selected features, tumors with high KRAS VAF exhibited lower 10th percentile values on the V_e_ map, indicating a tendency towards decreased signal intensity of the tumor. Additionally, a higher value of size zone nonuniformity normalized (SNZZ) on the K_ep_ map suggested the presence of heterogeneous zone size volumes in high KRAS VAF tumors. These findings imply that the DCE-parameter-based radiomics model has the potential to capture genetic or radiologic characteristics of tumors.

There are several limitations to be noted regarding this study. First, the number of patients in the cohort was small. This prospective study explored genetic information and DCE MRI, which are not routinely obtained during the management of PDAC patients. Therefore, having a small number of participants was inevitable. Second, we could not perform external validation using public or outside data. It was difficult to find publicly available data on patients, including DCE MRIs of the pancreas and genetic information. Further prospective studies in other hospitals may be necessary to generalize the results of this study. Third, we could not evaluate the impact of KRAS VAF on patient survival. As some research has shown that a high KRAS VAF is associated with poor patient outcomes, it would be better to evaluate the impact of the KRAS VAF or DCE radiomics model on survival outcomes in our cohort. However, this was impossible because variable treatment methods were applied to the patients in this study. This issue should be solved with a further study involving patients who undergo homogeneous treatment. Fourth, the interobserver agreement for rim enhancement was good, albeit relatively low (κ = 0.670). In MRI research, it has been reported as 0.85, whereas in CT research it was 0.64 and 0.766 (4, 40, 41). We speculate that differences in imaging modality can cause the differences in interobserver agreement. Even though we used to consensus results to reduce the variability between radiologists, further studies with more readers with different imaging modality would be helpful to generalize the current results.

In conclusion, rim enhancement of PDAC is associated with KRAS VAF among NGS-based genetic information. For predicting the KRAS VAF status in PDAC, a radiomics model based on DCE maps showed promising results.

## Data availability statement

The original contributions presented in the study are included in the article/**Supplementary Material**, further inquiries can be directed to the corresponding authors.

## Ethics statement

The studies involving humans were approved by The institutional review board of Eunpyeong St. Mary’s hospital. The studies were conducted in accordance with the local legislation and institutional requirements. The participants provided their written informed consent to participate in this study.

## Author contributions

MC: Writing – review & editing, Writing – original draft, Visualization, Investigation, Funding acquisition, Formal analysis, Data curation, Conceptualization. SY: Writing – review & editing, Writing – original draft, Supervision, Methodology, Investigation, Formal analysis, Data curation, Conceptualization. YL: Writing – review & editing, Formal analysis, Conceptualization. EJ: Writing – review & editing, Formal analysis, Data curation. SP: Writing – review & editing, Software, Resources. DH: Writing – review & editing, Software, Resources. DN: Writing – review & editing, Software, Resources.
