# Supplementary material for: Rim enhancement of pancreatic ductal adenocarcinoma: investigating the relationship with DCE-MRI-based radiomics and next-generation sequencing
Source: Front Oncol. 2024 Mar 8;14:1304187. doi: 10.3389/fonc.2024.1304187 (PMC10959187; doi:10.3389/fonc.2024.1304187)
Supplement: Supplementary file 1 [file DataSheet_1.pdf]

Rim enhancement of pancreatic ductal adenocarcinoma: Investigating the relationship with DCE-MRI-based radiomics and next-generation sequencing

Supplementary Table E1. MRI sequences and parameters

	**Heavily T2WI**	**T2WI**	**DWI**	**DCE-MRI**	**Delayed CE T1WI**
**Planes**	Axial, coronal	Axial	Axial	Axial	Coronal, sagittal
**Sequence**	HASTE	TSE	EPI	GRASP	CAIPIRINHA-VIBE
**TR (msec)**	900	2520	7100	3	3.7
**TE (msec)**	130	95	48	1.58	1.23
**Flip angle (°)**	135	120	90	9	10
**Thickness (mm)**	4	4	4.5	3	2.5
**Interslice gap (mm)**	1	1	0.9	0	0
**Resolution (mm^2)**	0.7 × 0.7	1.2 × 1.2	1.6 × 1.6	1.4 × 1.4	0.8 × 0.8
**Field of view (mm)**	400 × 325	400 × 400	400 × 320	400 × 400	300 × 400
**NEX**	1	1	1	1	1
**B values (s/mm^2)**	-	-	0, 50, 400, 800, 1000	-	-
**Acquisition time (min:sec)**	0:42	1:36	4:49	5:48	0:14

TR, repetition time; TE, echo time; NEX, number of excitations; T2WI, T2-weighted imaging; T1WI, T1-weighted imaging; DCE, dynamic contrast-enhanced; DWI, diffusion-weighted imaging; CE T1WI, contrast-enhanced T1-weighted imaging; HASTE, Half-Fourier Acquisition Single-shot Turbo Spin–echo; TSE, Turbo spin echo; EPI, Echo Planar Imaging; GRASP, Golden-angle RAdial Sparse Parallel MRI; CAIPIRINHA, Controlled Aliasing In Parallel Imaging Results In Higher Acceleration; VIBE, Volumetric Interpolated Breath-hold Examination

Supplementary Figure E1. Cluster map of the radiomics features in dynamic contrast-enhanced parametric maps in different patients

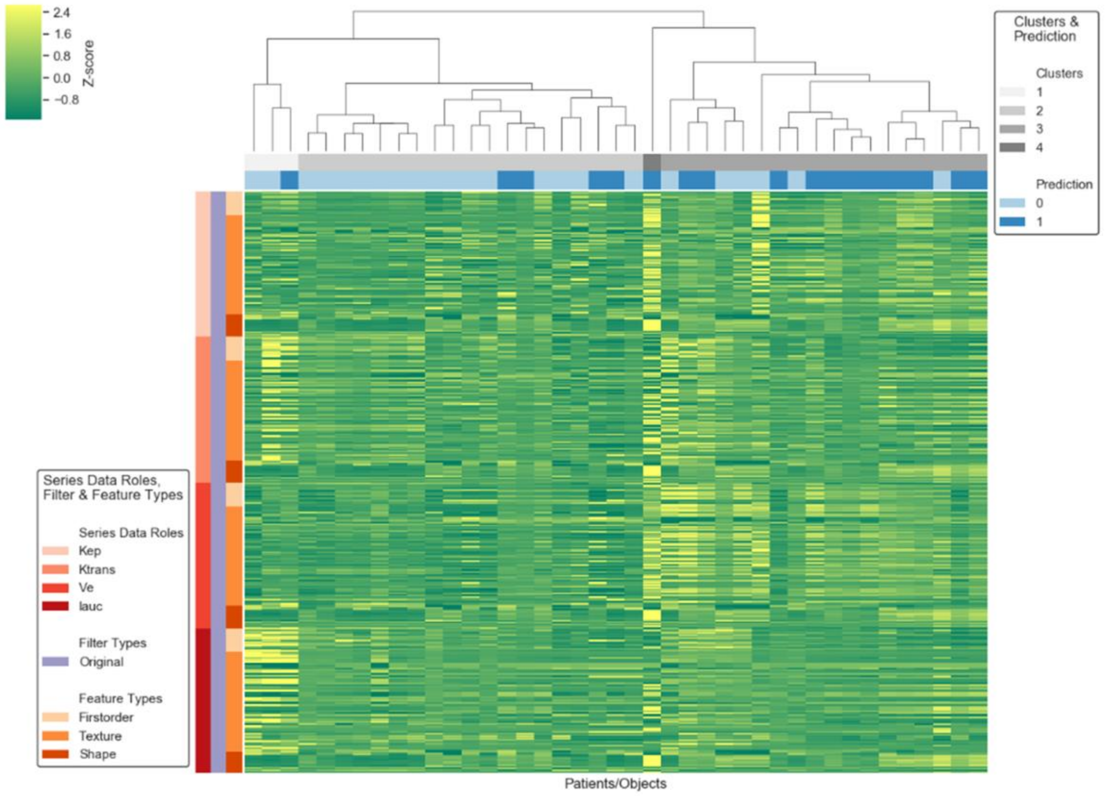

Supplementary Figure E2. Mutation profile of pancreatic ductal adenocarcinoma in this study

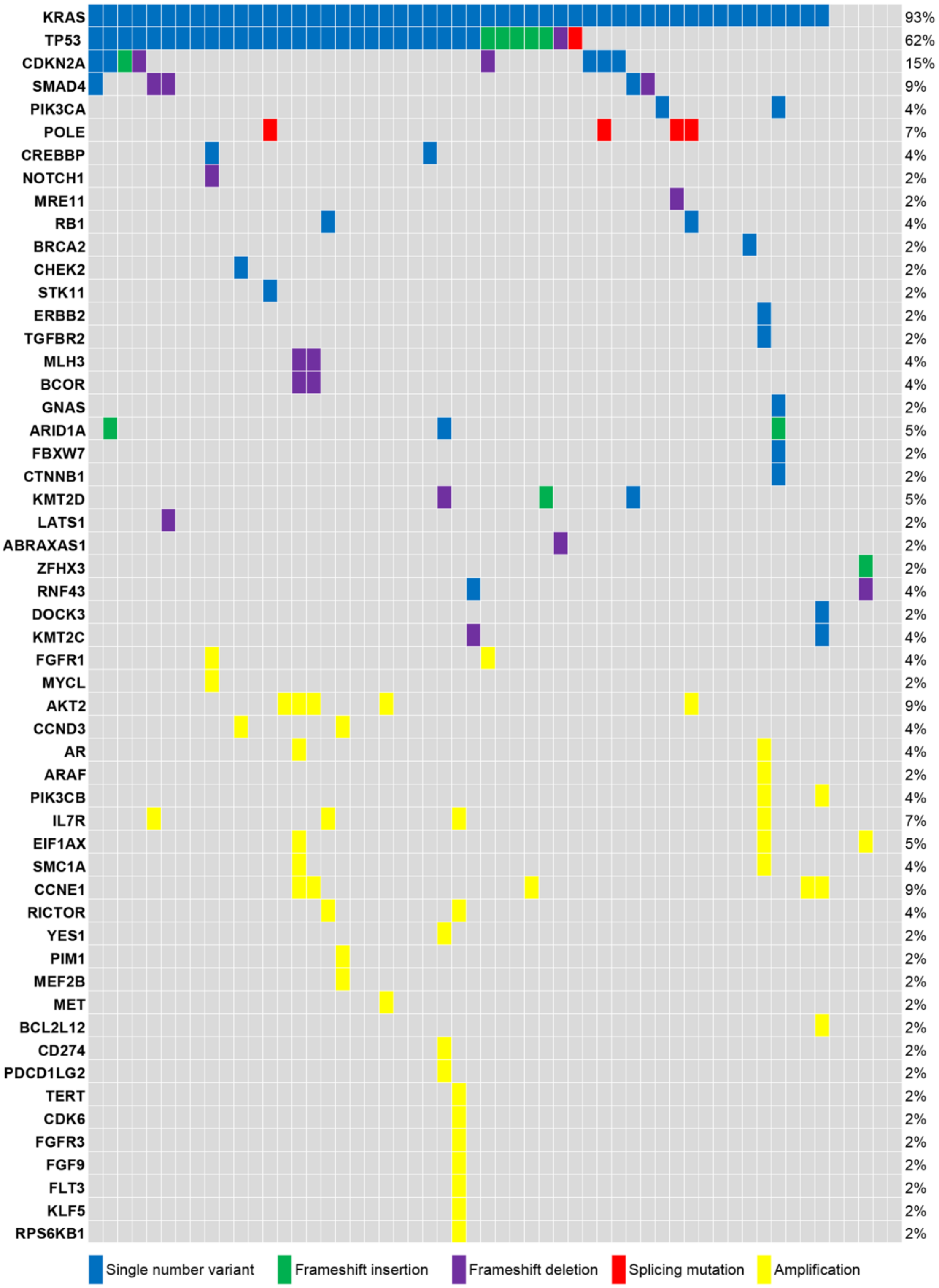

Supplementary Figure E3. ROC curves of tumor size (a, b) and KRAS variant allele frequency (c, d) for discriminating tumors with and without rim enhancement in the training set and test set, respectively

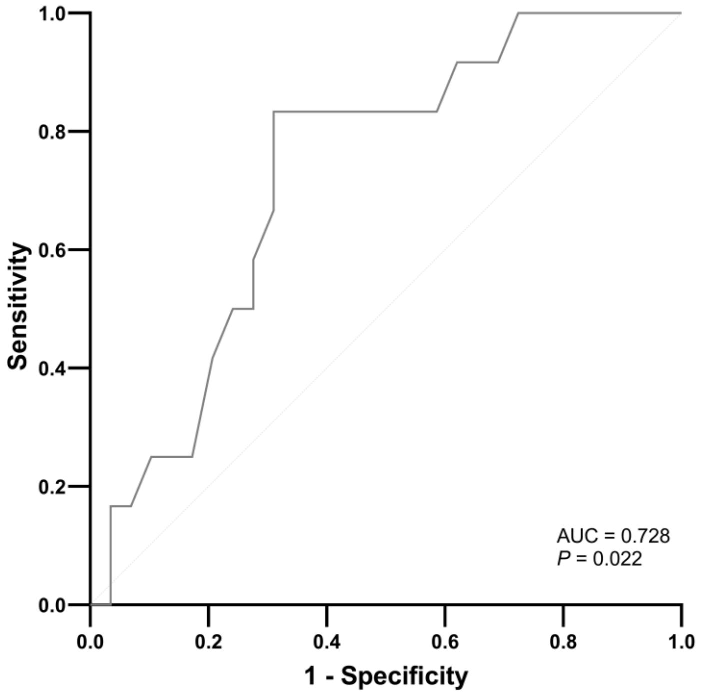

(a)

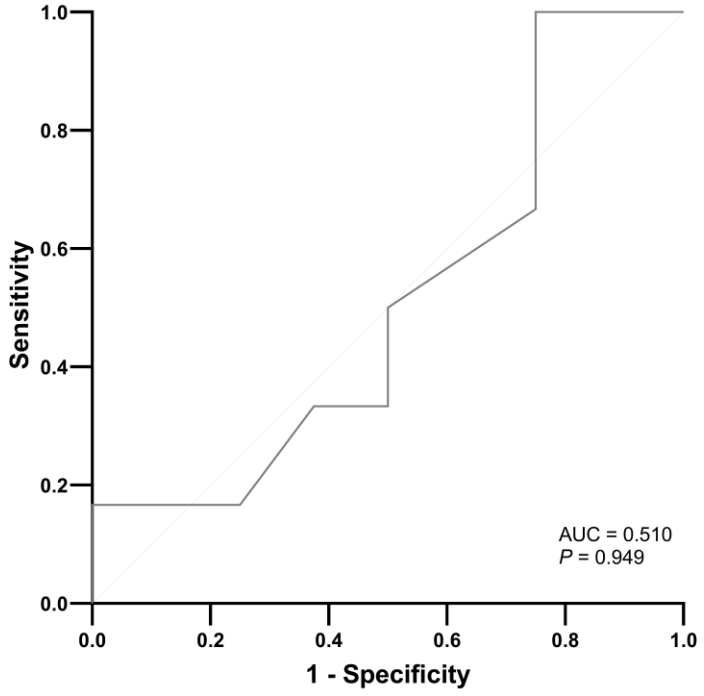

(b)

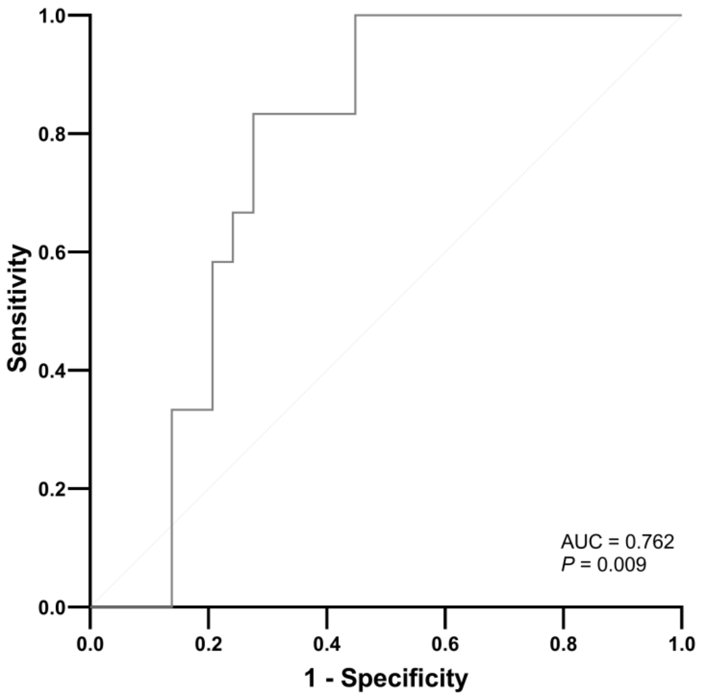
Sensitivity
1 - Specificity
AUC = 0.762
P = 0.009
(c)

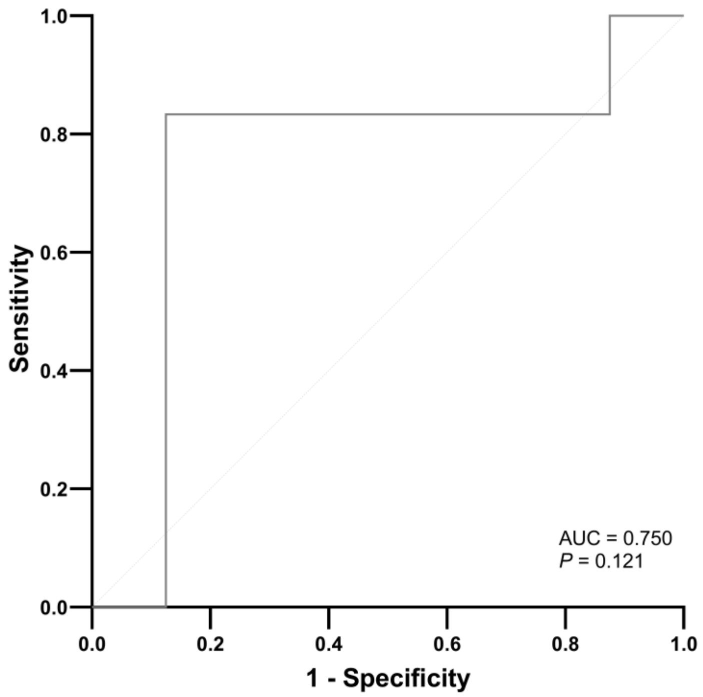
Sensitivity
1 - Specificity
AUC = 0.750
P = 0.121
(d)

Supplementary Figure E4. Heatmap of the 10 most relevant radiomics features for differentiating between tumors with low and high *KRAS* variant allele frequencies

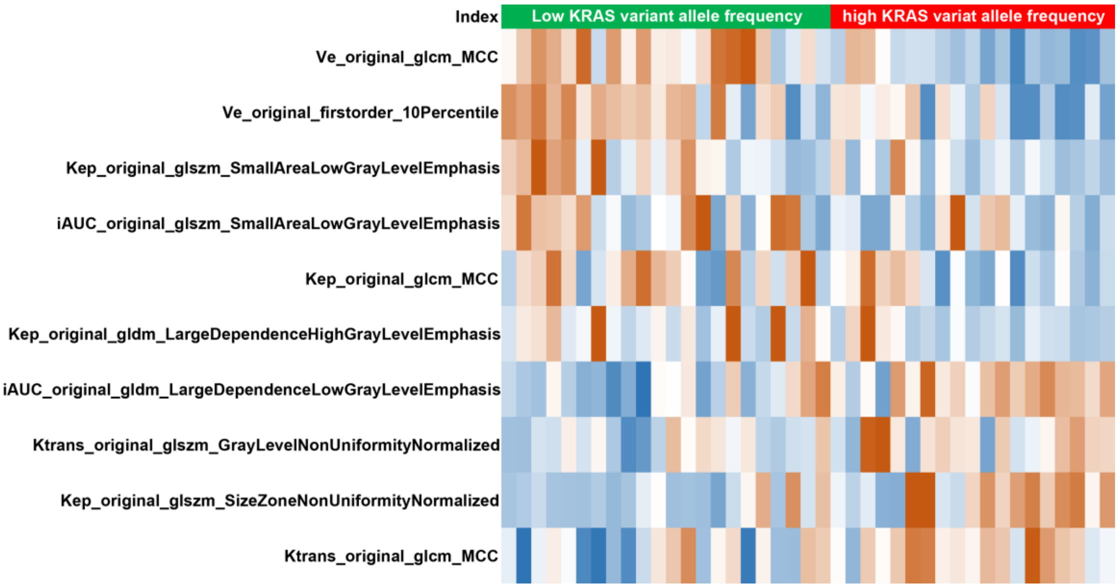

GLCM, Gray Level Co-occurrence Matrix; GLDM, Gray Level Dependence Matrix; GLSZM, Gray Level Size Zone Matrix; MCC, Maximal Correlation Coefficient
